# A Rare Case of Deglutition-Induced Atrial Fibrillation Secondary to Esophageal Dilation

**DOI:** 10.7759/cureus.58497

**Published:** 2024-04-17

**Authors:** Kathan Trivedi, Raymond Munoz

**Affiliations:** 1 Internal Medicine, Methodist Health System, Dallas, USA

**Keywords:** tachy-arrhythmia, propafenone, atrial fibrillation, esophageal dilation, deglutition

## Abstract

Atrial fibrillation (AF) is the most common arrhythmia in the world. However, deglutition-induced tachyarrhythmias are exceptionally rare. Diagnosis relies on a documented history, Holter monitoring and echocardiograms. The mechanism underlying deglutition-induced tachycardia remains uncertain, with leading hypotheses suggesting mechanical left atrial stimulation after esophageal distention or activation of the vagus nerve due to increased intra-esophageal pressure. Lifestyle changes, medications (e.g., beta-blockers and antiarrhythmics), and radiofrequency catheter ablation are viable treatment options. First-line treatment is usually beta-blockers, but they have limited effectiveness due to the poorly understood mechanisms behind this pathological condition. Sodium channel blockers targeting vagal motor fibers decrease esophageal muscle contraction force by reducing axonal transmission, supporting the theory that inhibiting rapid sodium channels may mitigate atrial tachycardias. This mechanism presents a promising approach for managing deglutition-induced atrial fibrillation. We present a unique case of a 58-year-old female diagnosed with deglutition-induced atrial fibrillation secondary to esophageal dilation who was successfully treated with the antiarrhythmic propafenone, supporting the vagus nerve hypothesis.

## Introduction

Atrial fibrillation (AF) is the most common supraventricular arrhythmia that interrupts normal cardiac function [1]. Although AF has several etiologies, deglutition-induced tachyarrhythmias are quite rare [2]. Patients with deglutition-induced AF experience intermittent palpitations that are consistent and reproducible with swallowing events. Among patients who present with paroxysmal atrial arrhythmias, only 0.6% of the known cases have been deglutition-induced [3,4]. The pathophysiology of the condition remains poorly understood due to its rarity.

Currently, a deglutition-induced AF diagnosis depends on a reliable and consistent history as well as the exclusion of other etiologies of tachyarrhythmias. Tools such as Holter monitors and echocardiograms are helpful to assess for arrhythmias and potential structural abnormalities, respectively. Initial management includes lifestyle changes, such as avoiding foods that induce tachyarrhythmia [5]. Pharmacologic options include beta-blockers, calcium channel blockers, and antiarrhythmic medications for the treatment of symptoms [3]. Radiofrequency catheter ablation is used as a permanent solution for those with refractory cases and is very useful when a single focus has been identified [3]. We present the case of a 58-year-old female with complaints of frequent palpitations and ultimately diagnosed with deglutition-induced AF secondary to esophageal dilation.

## Case presentation

A 58-year-old Caucasian female presented with complaints of a two-week-long history of short-lived palpitations that dissipated within one to two minutes from onset. These palpitations were not associated with new-onset chest pain, dyspnea, paroxysmal nocturnal dyspnea, orthopnea, or new-onset lower extremity edema. Notably, just prior to the initiation of the symptoms, she had undergone dilation of the esophagus at an outside hospital for esophageal strictures. The medical workup until the time of presentation was notable for an unremarkable exercise stress test with no ischemic electrocardiogram (EKG) changes or arrhythmia with stress or at baseline. She also had a low-risk stress echocardiogram, with a post-stress left ventricular ejection fraction of 80% and no regional wall motion abnormalities.

Her past medical history consisted of esophageal strictures (status: post-recent esophageal dilation), chronic immunosuppression (status: post-kidney transplant), hypertension, hyperlipidemia, and hypothyroidism. Her home medications included rosuvastatin 20 mg daily for hyperlipidemia as well as prednisone 5 mg daily and mycophenolate 360mg daily for the immunosuppressive regimen. She denied any smoking history, alcohol, or illicit drug use. At the time of presentation, her vital signs were within normal limits. Her physical exam was unremarkable, including the cardiac examination, which revealed normal S1 and S2 heart sounds in regular rhythm with no murmurs, rubs, or gallops. Hemoglobin and hematocrit were normal at the time of admission as well as the serum creatinine and thyroid-stimulating hormone levels. ​​ An admission EKG indicated a normal sinus rhythm with non-specific ST segment abnormalities and normal QTc. A subsequent chest x-ray revealed no evidence of cardiopulmonary disease.

**Figure 1:**
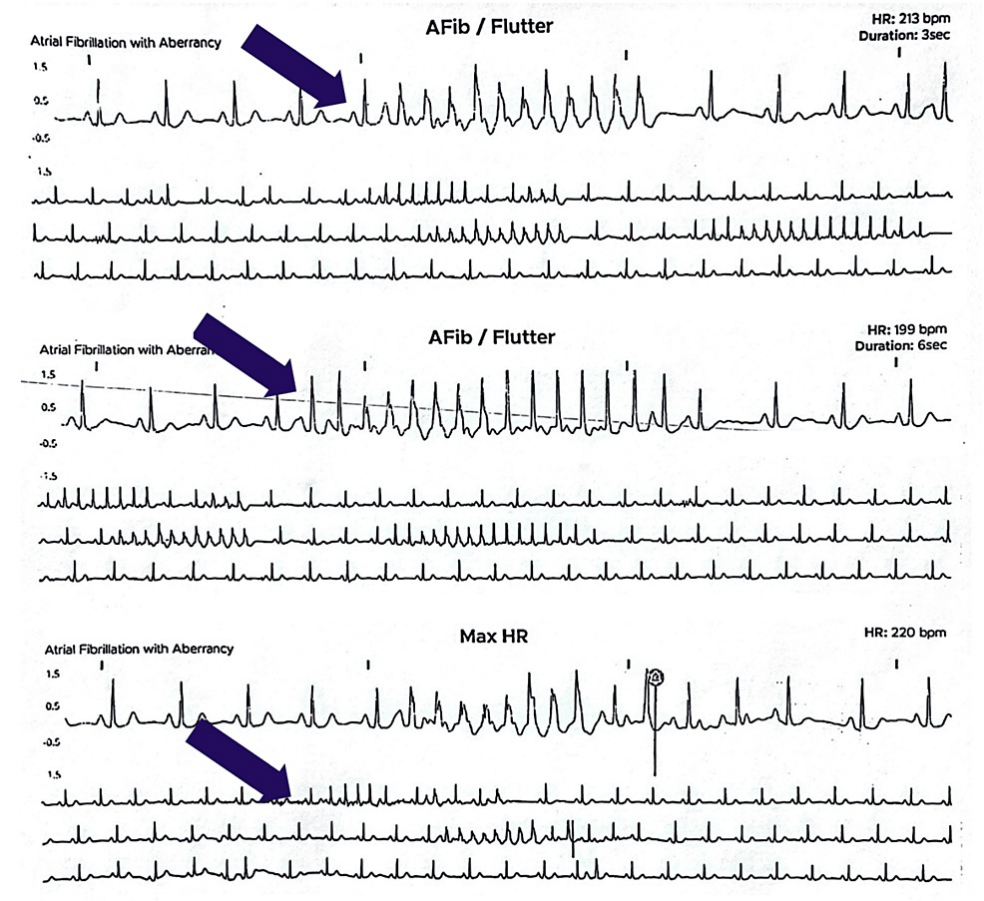
Holter monitoring readout showing multiple instances of suspected atrial fibrillation/flutter with aberrancy and atrial tachycardia as well as several premature atrial complexes during episodes of swallowing (indicated with arrows).

Given the benign presentation at the time of admission, the patient was discharged home with a 48-hour Holter monitor for further evaluation of possible arrhythmias. The patient was instructed to trigger an "event" during instances of palpitations. She followed up with the electrophysiology clinic in an outpatient setting to further assess the Holter monitor findings. The interrogation demonstrated a sinus rhythm and mild sinus bradycardia with short episodes of AF with a peak heart rate of 220 beats per minute. The episodes symptomatically lasted for approximately four to five seconds prior to spontaneously resolving. The maximum length of these episodes was 38 seconds (Figure 1). These episodes correlated directly to instances of swallowing by the patient, resulting in a diagnosis of deglutition-induced AF.

The patient was placed on metoprolol 25 mg twice daily and apixaban 5 mg twice daily. Though the intensity of further episodes varied, this new regimen did not eliminate the episodes. She returned to the emergency department due to persistent paroxysms of AF. Her heart rate-controlling agent has switched from metoprolol to propafenone 225 mg twice daily. In her next follow-up appointment thirty days later, she noted significant improvement in her symptoms and denied any further episodes of palpitations. However, she complained of dizziness and weakness, which were attributed to propafenone side effects. At this time, propafenone was transitioned to flecainide 75 mg twice daily, which resulted in the complete resolution of her symptoms.

## Discussion

Deglutition-induced supraventricular tachycardia is an exceedingly rare phenomenon, representing less than 1% of reported AF cases. While the first case was noted in 1926, to this day, less than 100 reports exist of such a phenomenon [6]. Hence, the literature regarding the mechanism and treatment options is lacking.

**Figure 2:**
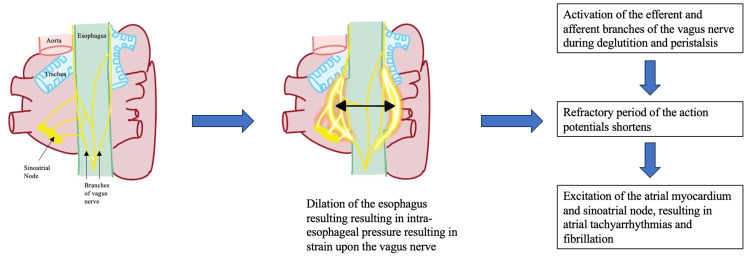
Suspected mechanism of action for atrial fibrillation secondary to esophageal dilation in our patient. Note: This image is the author's own creation.

Currently, there are several leading hypotheses about the mechanisms underlying deglutition-induced supraventricular tachycardia. The first proposed mechanism is that arrhythmias are induced by mechanical stimulation of the left atrium after undergoing distention of the esophagus. In fact, a study conducted by Cohen and colleagues showed the reproduction of AF with physical balloon dilation of the esophagus near the level of the left atrium, similar to our patient, who underwent a recent esophagogastroduodenoscopy [7]. In fact, other pathophysiologies involving esophageal dilation, including achalasia, which results in failure of the lower esophageal sphincter to relax, have also been linked to deglutition-induced AF [8]. A later study by Kalloor and associates showed successful treatment with a circular esophageal myotomy, which is often a treatment used to treat achalasia [9]. A second proposed mechanism involves activation of the efferent and afferent branches of the vagus nerve from increased intra-esophageal pressure, which was the likely etiology in this patient (Figure 2) [10]. As the afferent and efferent vagal reflexes are activated with primary peristalsis from swallowing or esophageal dilation, the stimulation can shorten the refractory period, resulting in a nonuniform excitation of the atrial myocardium and atrial tachycardia [11,12]. Lastly, a final hypothesis involves activation of the sympathetic nervous system, which could result in nonuniform atrial depolarization and trigger AF [13].

The treatment of deglutition-induced supraventricular tachycardia varies but is often quite similar to the treatment of paroxysmal AF. The first attempts to treat a patient with deglutition-induced supraventricular tachycardia are usually with rate-controlling beta-blockers and anticoagulation. Studies have shown that while beta-blockers are often the most commonly used medication, they are often the least effective, with only approximately 18% of patients experiencing resolution of symptoms [14]. This was noted in our patient, who continued to experience palpitations despite strict adherence to metoprolol.

A sustained-release formulation of propafenone, a class IC antiarrhythmic that performs its action mainly by blocking rapid sodium channels, terminates approximately 83% of supraventricular tachyarrhythmias [15]. This success was demonstrated in our patient, who experienced resolution of symptoms with propafenone and flecainide. Given the success of these medications, this case strengthens the second proposed mechanism involving the activation of branches of the vagus nerve from increased intra-esophageal pressure. In fact, recent studies have demonstrated that sodium channel inhibitors that act on the axons of vagal motor fibers cause a decrease in the force of esophageal striated muscle contraction evoked by electrical stimulation of the motor fibers [16]. As such, the number of motor axons conducting action potentials in the presence of sodium inhibitors is significantly decreased, causing a decrease in the contraction force. This finding strengthens the theory that blocking rapid sodium channels may reduce excitation of the atrial myocardium and, subsequently, atrial tachycardias by reducing the force of the esophageal muscle contraction. While propafenone and flecainide were previously used with hesitancy due to misconceptions of an increased risk of ventricular proarrhythmia, recent studies have shown that such adverse events occur in less than 1% of the population. Furthermore, Echt and Ruskin demonstrated 97% effectiveness for the acute termination of AF with such antiarrhythmics, while noting a favorable safety profile when administered to patients with minimal or no structural heart disease [17].

Other case reports have noted successful treatment and resolution of symptoms with other antiarrhythmic medications, including digoxin, quinidine, and procainamide [18]. Ultimately, if pharmacological treatments were unsuccessful, radiofrequency ablation was noted to be a permanent therapeutic option with nearly a 100% success rate [19].

## Conclusions

This report describes one of the only known cases of a patient diagnosed with deglutition-induced AF secondary to esophageal dilation who was managed effectively with class IC antiarrhythmic medications. The efficacy of class 1c antiarrhythmic in this context provides further credence to the hypothesis that the inhibition of rapid sodium channels may attenuate the excitation of the atrial myocardium, consequently mitigating atrial tachycardias. By reducing the force exerted by esophageal muscle contractions, these antiarrhythmics highlight a promising avenue for the management of deglutition-induced AF.
